# Q1020R in the spike proteins of MERS-CoV from Arabian camels confers resistance against soluble human DPP4

**DOI:** 10.1128/jvi.00282-26

**Published:** 2026-04-06

**Authors:** Nianzhen Chen, Hannah Kleine-Weber, Khaled Alkharsah, Michael Winkler, Asisa Volz, Marcel A. Müller, Victor M. Corman, Christian Drosten, Markus Hoffmann, Stefan Pöhlmann

**Affiliations:** 1 German Primate Center – Leibniz Institute for Primate Research, Göttingen, Germany; 2 Faculty of Biology and Psychology, Georg-August University Göttingen 98900 , Göttingen, Germany; 3 Institute of Molecular Virology, Ulm University Medical Center 27197, Ulm, Germany; 4 Department of Microbiology, College of Medicine, Imam Abdulrahman Bin Faisal University (IAU) 48135, Dammam, Saudi Arabia; 5 Institute of Virology, University of Veterinary Medicine Hannover 26556 , Hanover, Germany; 6 German Center for Infection Research, Partner Site Hannover-Braunschweig 574554 , Hannover, Germany; 7 Institute of Virology, Charité - Universitätsmedizin Berlin, corporate member of Freie Universität Berlin, Humboldt-Universität zu Berlin, and Berlin Institute of Health 522475 , Berlin, Germany; 8 German Centre for Infection Research (Deutsches Zentrum für Infektionsforschung), Partner site Charité, Berlin, Germany; 9 Labor Berlin-Charité Vivantes GmbH 646268 , Berlin, Germany; 10 German Center for Infection Research (DZIF), associated partner site Göttingen, Göttingen, Germany; Loyola University Chicago - Health Sciences Campus, Maywood, Illinois, USA

**Keywords:** Middle East respiratory syndrome, spike, DPP4, soluble DPP4

## Abstract

The Middle East respiratory syndrome coronavirus (MERS-CoV) is a pre-pandemic coronavirus that is transmitted from camels, the natural reservoir, to humans and can cause severe disease. MERS cases have been documented in Arabia but not Africa, although the virus is circulating in both Arabian and African camels. Further, evidence has been provided that viruses in African camels might have a reduced capacity to cause disease. However, the underlying determinants are incompletely understood. Here, employing pseudotyped particles as model systems for MERS-CoV entry into cells, we compared cell entry of viruses from African and Arabian camels and its inhibition. We show that viruses found in Arabian camels and recent human cases are less susceptible to inhibition by human soluble DPP4 (sDPP4) than viruses from African camels, although both enter human cells efficiently and are comparably sensitive to inhibition by interferon-induced transmembrane (IFITM) proteins and neutralizing antibodies. Furthermore, relative resistance to sDPP4 was linked to mutation Q1020R, present in the spike proteins of recent Arabian but not African viruses. Finally, indirect evidence was obtained that sDPP4 in human plasma can inhibit MERS-CoV cell entry. These results support the concept that soluble DPP4 might constitute a natural barrier against human infection that is more efficiently overcome by viruses currently circulating in Arabian camels than those in African camels.

**IMPORTANCE:**

Middle East respiratory syndrome coronavirus (MERS-CoV) is an emerging virus that can cause severe lung disease, MERS, and is transmitted from camels to humans. Although MERS-CoV infects camels in both Africa and Arabia, MERS cases have only been documented in Arabia for reasons that remain incompletely understood. Here, we provide evidence that viruses recently circulating in Arabian camels and causing human infections are less susceptible to inhibition by human soluble DPP4 (sDPP4)—a secreted version of the viral receptor that is present in various bodily fluids. Furthermore, we link sDPP4 resistance to mutation Q1020R in the spike protein of these viruses. These results suggest that viruses currently circulating in Arabian camels are better equipped to overcome a natural barrier to infection, sDPP4, than those circulating in African camels.

## INTRODUCTION

Middle East respiratory syndrome coronavirus (MERS-CoV) is a zoonotic virus transmitted from dromedary camels to humans that causes severe disease with a case fatality rate exceeding 30%. MERS-CoV circulates in both Arabian (clade A and B viruses) and African (clade C viruses) dromedary camel populations. However, despite serological and virological evidence of camel-to-human transmission in Africa, none of the over 2,600 confirmed human MERS cases reported to date have originated from the continent (1–3). Recent cases have been attributed primarily to clade B, lineage five viruses, which exhibit enhanced replicative fitness (4–6) and have predominated since 2015 (7, 8). In contrast, clade C viruses appear to be attenuated (6, 9). Nonetheless, the molecular determinants underlying the differences in replicative capacity and virulence among MERS-CoV strains remain incompletely understood.

The MERS-CoV spike (S) protein facilitates viral entry into target cells, determines viral cell and species tropism, and is the primary target of the neutralizing antibody response. For cell entry, the receptor-binding domain (RBD) within the S1 subunit of the S protein binds to the cellular receptor, DPP4/CD26 (10–13). Subsequently, the S2 subunit facilitates fusion of the viral and cellular membranes. In order to obtain membrane fusion competence, the S protein depends on activation by host cell proteases. Upon pre-cleavage of the S protein by furin in infected cells (14–18), the serine protease TMPRSS2 cleaves and thereby activates the S protein during viral entry into cells, and its activity is essential for full viral spread and pathogenesis in animal models (19–22). Alternatively, the pH-dependent cysteine protease cathepsin L can activate the S protein in host cell endo/lysosomes (18–20), but its role in viral spread and pathogenesis is less clear.

Several factors can negatively regulate viral entry: interferon-induced transmembrane (IFITM) proteins block cathepsin L-dependent entry of MERS-CoV (23, 24), while plasminogen activator inhibitor 1 (PAI-1) blocks TMPRSS2 activity and interferes with influenza A virus (25) and possibly also MERS-CoV proteolytic activation. Further, the cellular protein adenosine deaminase (ADA) blocks cell entry of MERS-CoV by binding to DPP4, thereby blocking S protein engagement of its receptor (26). In addition, DPP4 can be shed from the cell surface (27–29), which might require activity of metalloproteinases (30), and soluble DPP4 (sDPP4) can block MERS-CoV entry into cultured cells and viral spread in experimentally infected animals (31, 32). Finally, neutralizing antibodies from convalescent patients inhibit MERS-CoV cell entry, mainly by preventing the binding of the RBD to DPP4 (1, 33–36).

Here, we investigated whether African and Arabian MERS-CoV differ in their capacity to enter cells and/or are differentially sensitive to negative regulators of the entry process. We provide evidence that viruses from Saudi Arabian camels are less sensitive to sDPP4 than their African counterparts and show that a signature mutation in the S protein of the Arabian viruses, Q1020R, contributes to this phenotype.

## RESULTS

**Fig 1:**
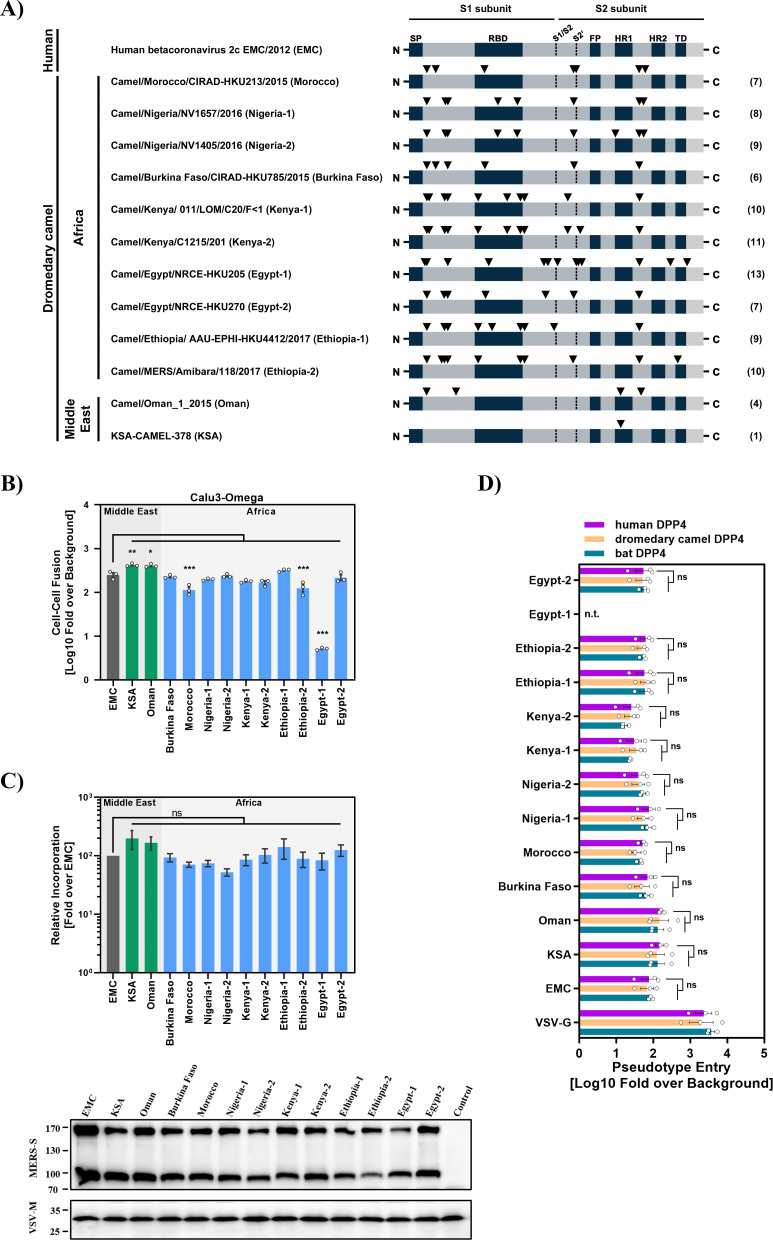
Virion incorporation, cell-cell fusion, and DPP4 usage of the MERS-CoV S proteins analyzed. (**A**) Domain organization of the MERS-CoV S protein and mutations (black arrowheads) found in viruses from African and Arabian dromedary camels compared with the human betacoronavirus 2c EMC/2012 (EMC) S protein. Abbreviations: SP = signal peptide, S1/S2 and S2` = cleavage sites, RBD = receptor binding domain, FP = fusion peptide, HR1/HR2 = heptad repeat 1/2, TD = transmembrane domain. (**B**) For analysis of cell-cell fusion, 293T effector cells coexpressing the indicated S proteins (or no S protein) along with the beta-galactosidase alpha fragment were co-cultured with target cells (Calu-3) stably expressing the beta-galactosidase omega fragment (Calu3-Omega). At 24 h post-cocultivation, beta-galactosidase activity was measured in cell lysates. The average data from three independent biological replicates are presented in panels A and B, each performed in technical quadruplicates. Data were normalized against fusion measured in the absence of S protein. Error bars indicate the SEM. Statistical significance of differences between EMC spike and the other S proteins studied was assessed by one-way analysis of variance (ANOVA) with Dunnett’s posttest (*P* > 0.05, not significant [ns]; *P* ≤ 0.05, *; *P* ≤ 0.01, **; *P* ≤ 0.001, ***). (**C**) For analysis of particle incorporation of the S proteins under study, vesicular stomatitis virus-based pseudotypes (VSVpp) harboring the indicated S proteins were concentrated by centrifugation and analyzed by Western Blot for efficiency of S protein incorporation using an anti-V5 antibody. Detection of VSV-M served as loading control. The results of a representative blot are shown in the lower panel. Upper panel: Immunoblots (*n* = 7) were quantified using ImageJ software. Combined S protein signals (uncleaved [S0] and cleaved [S2]) were normalized against the corresponding signal of the loading control (VSV-M). Mean values are shown, error bars indicate the standard error of the mean (SEM). Statistical significance of differences in particle incorporation efficiency between human betacoronavirus 2c EMC/2012 (EMC) and the other S proteins analyzed was determined by one-way ANOVA with Dunnett’s posttest (*P* > 0.05, not significant [ns]; *P* ≤ 0.05, *; *P* ≤ 0.01, **; *P* ≤ 0.001, ***). (**D**) In order to analyze usage of DPP4 for cell entry, BHK-21 cells transiently expressing DPP4 of human, dromedary camel, or bat origin were inoculated with pseudotypes bearing the indicated S proteins or VSV-G. At 16 h post-inoculation, pseudotype entry was measured by quantifying luciferase activity in cell lysates and normalized against particles without viral envelope protein. The average of three independent experiments (each performed with four technical replicates) is shown. Error bars indicate the SEM. Statistical significance was tested by two-way ANOVA with Dunnett’s posttest (*P* > 0.05, not significant [ns]; *P* ≤ 0.05, *; *P* ≤ 0.01, **; *P* ≤ 0.001, ***). n.t., not tested.

We investigated whether the S proteins of viruses from African (*n* = 10) and Arabian (*n* = 2) camels exhibit distinct biological properties, employing a previously described cell-cell fusion assay and pseudotyped viral particles (37, 38). The S protein of MERS-CoV EMC/2012 (EMC, obtained from a human case) served as reference. The S proteins studied differed in up to 13 mutations located in several domains, including the RBD and heptad repeat 1 (HR1) (Fig. 1A; Fig. S1). All S proteins drove robust fusion of 293T effector cells with Calu-3 target cells (Fig. 1B). The only exception was the S protein of Egypt-1, which was inactive due to a mutated cleavage site, as expected (39). Furthermore, all S proteins were efficiently cleaved and incorporated into particles (Fig. 1C), enabling a direct comparison for host cell entry and its inhibition. Finally, all S proteins except Egypt-1 were able to drive robust entry into BHK21 cells transfected to express human or camel DPP4 (Fig. 1D), consistent with expectations. In summary, all S proteins studied were efficiently expressed and were able to efficiently drive both cell-cell and virus-cell fusion. The S protein of Egypt-1 was inactive, as expected, but was included as a negative control in our further analyses.

### The S proteins of Arabian and African viruses mediate efficient entry into human cell lines and exhibit comparable dependence on activating proteases

**Fig 2:**
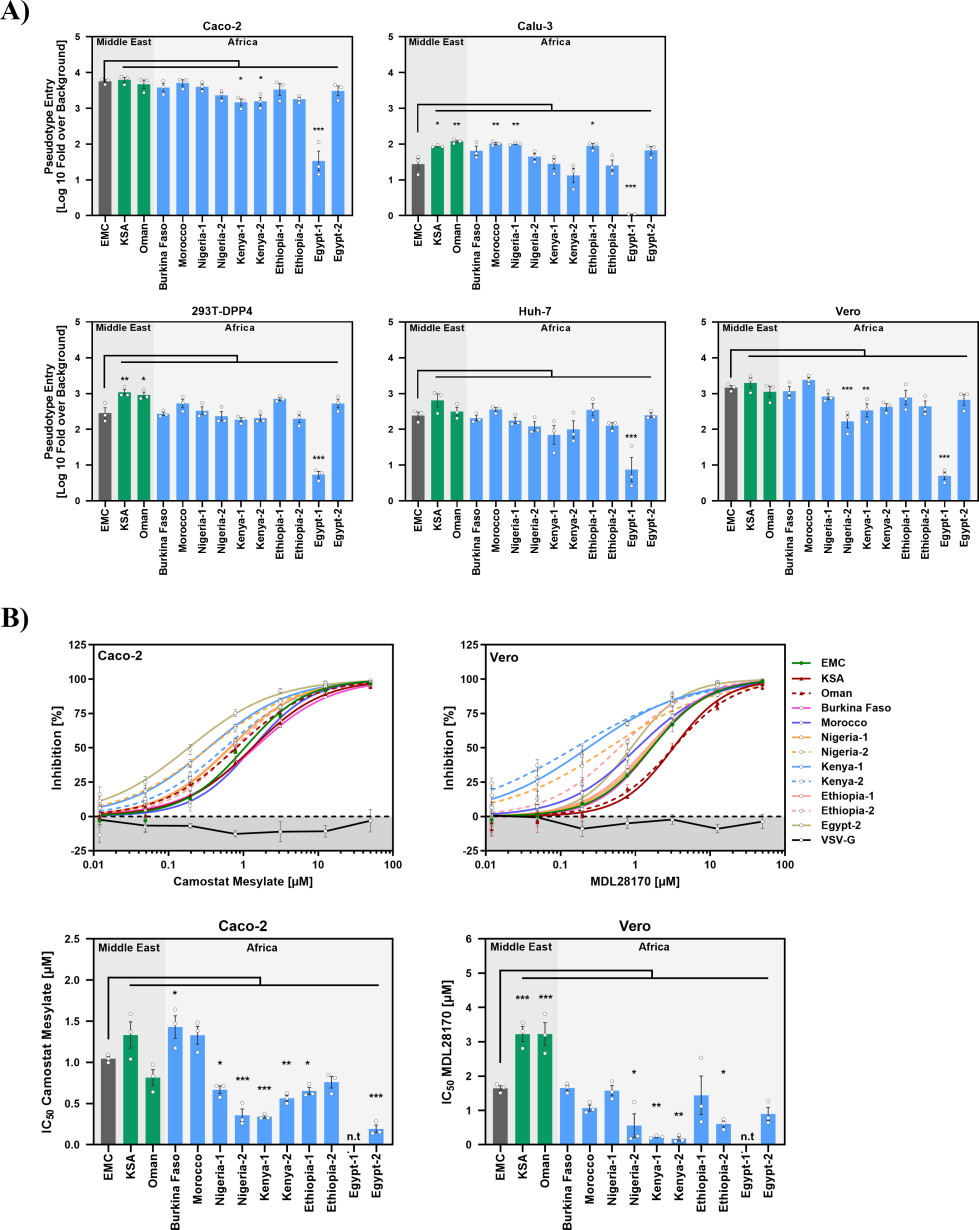
Virus-cell fusion driven by the S proteins of African and Arabian MERS-CoV is efficient and sensitive to inhibitors of cathepsin L and TMPRSS2. (**A**) To analyze virus-cell fusion, pseudotyped particles harboring the indicated S proteins were added onto the indicated cell lines and efficiency of cell entry was quantified by measuring the activity of pseudovirus-encoded luciferase in cell lysates at 16-18 h post transduction. The normalized average data from three independent biological replicates (each performed in technical quadruplicates) are presented, in which cell entry was normalized to particles lacking viral glycoproteins (set as 1). Error bars indicate the SEM. Statistical significance of differences between EMC spike and the other S proteins studied was assessed by one-way ANOVA with Dunnett’s posttest (*P* > 0.05, not significant [ns]; *P* ≤ 0.05, *; *P* ≤ 0.01, **; *P* ≤ 0.001, ***). (**B**) Protease dependence of viral entry was analyzed by preincubating Caco-2 and Vero76 cells for 1 h with varying concentrations (0 [DMSO], 0.049, 0.012, 0.195, 0.781, 3.125, 12.5, or 50 μM) of Camostat mesylate or MDL28170, respectively, before being inoculated with pseudotyped particles harboring the indicated S proteins. At 16–18 h postinoculation, pseudovirus entry efficiency was determined by quantifying luciferase activity in cell lysates. Entry into DMSO-treated cells was set as 100% (= 0% inhibition). Data in the top panels represent the mean of three biological replicates (each performed with four technical replicates). Based on this, the inhibitor concentration leading to half-maximal inhibition of pseudovirus entry (IC_50_) was calculated and is shown in the bottom panels. Error bars represent the SEM. Statistical significance between EMC and variant was evaluated by one-way ANOVA with Dunnett’s posttest (*P* > 0.05, not significant [ns]; *P* ≤ 0.05, *; *P* ≤ 0.01, **; *P* ≤ 0.001, ***). n.t., not tested.

We next investigated whether the robust entry into cells transfected to express DPP4 translated into efficient entry into cell lines endogenously expressing DPP4. For this, we employed 293T (kidney, human) cells stably expressing human DPP4 as well as Huh-7 (liver, human), Vero cells (kidney, African green monkey), Caco-2 (intestine, human) and Calu-3 (lung, human) cells as targets. All S proteins except Egypt-1 mediated efficient entry into the cell lines tested (Fig. 2A). Minor differences were detected but were not consistently observed among all cell lines studied. Entry driven by the S proteins of the Arabian viruses KSA and Oman was generally efficient, while some of the S proteins of African viruses mediated entry into Vero cells with reduced efficiency (Fig. 2A). Finally, all S protein-bearing particles were sensitive to inhibitors of the S protein-activating host cell proteases TMPRSS2 (Camostat) and Cathepsin L (MDL 28170) (Fig. 2B), although some differences were observed. For instance, particles bearing the KSA and Oman S proteins showed decreased MDL 28170 but similar camostat sensitivity as compared with control particles bearing EMC S protein, while particles harboring Kenya-1 and Kenya-2 S proteins exhibited augmented MDL 28170 and camostat sensitivity for at present unclear reasons (Fig. 2B).

### Particles harboring the S proteins of African and Arabian viruses show similar temperature stability and sensitivity to IFITM proteins and neutralizing antibodies

**Fig 3:**
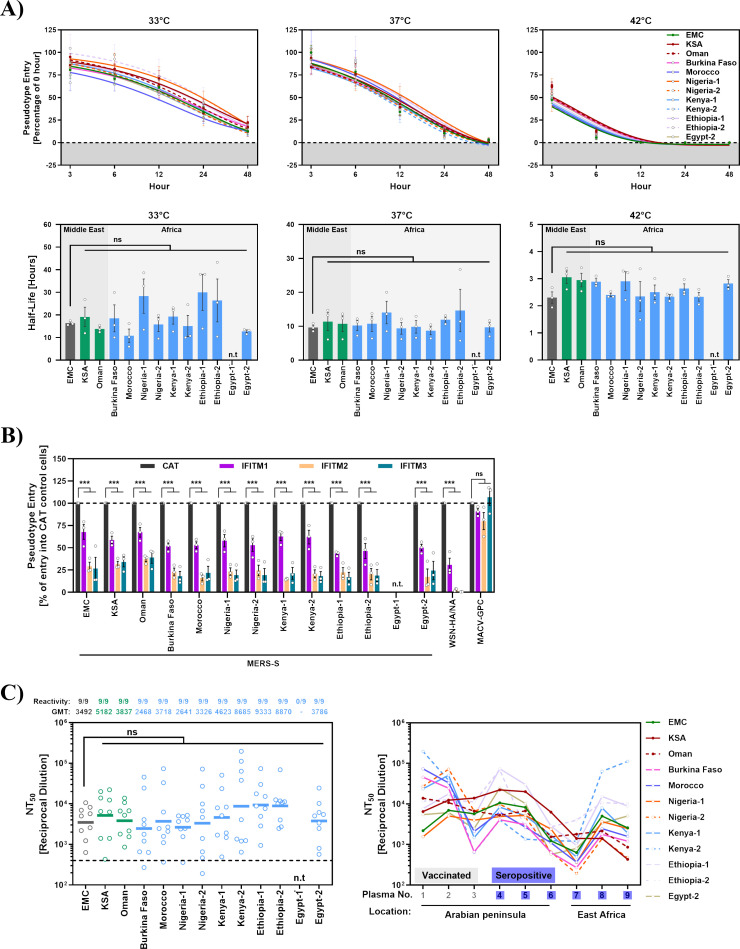
Particles pseudotyped with the S proteins of African and Arabian viruses are comparable in sensitivity to temperature, IFITM proteins, and neutralizing antibodies. (**A**) Pseudotyped particles harboring the indicated S proteins were incubated at 33°C, 37°C, or 42°C for different durations (0, 3, 6, 12, 24, and 48 h) before being added to Vero cells. At 16–18 h postinoculation, pseudovirus entry was measured by quantifying luciferase activity in cell lysates. Cell entry of particles pre-incubated for 0 h at the respective temperatures was set as 100% entry. The data represent the average of three independent experiments with four technical replicates each. Error bars indicate the SEM. The lower panels present the half-life derived from nonlinear curve fitting using the exponential decay model. Statistical significance was tested by one-way ANOVA with Dunnett’s posttest (*P* > 0.05, not significant [ns]; *P* ≤ 0.05, *; *P* ≤ 0.01, **; *P* ≤ 0.001, ***). n.t., not tested. (**B**) 293T cells stably expressing interferon-induced transmembrane proteins (IFITM1 to IFITM3) or chloramphenicol acetyltransferase (CAT; control) were transduced with pseudoviruses harboring the indicated S proteins, influenza A virus (WSN, subtype H1N1) hemagglutinin and neuraminidase (WSN-HA/NA), Machupo virus glycoprotein (MACV-GPC), or no viral glycoprotein (negative control; data not shown). At 18 h post-inoculation, efficiency of pseudovirus entry was analyzed by measuring luciferase activity in cell lysates. Entry into control cells, 293T-CAT, was set as 100%. Presented are the averages from three individual experiments performed with four technical replicates. Error bars indicate the SEM. Statistical significance was tested by two-way ANOVA with Dunnett’s posttest (*P* > 0.05, not significant [ns]; *P* ≤ 0.05, *; *P* ≤ 0.01, **; *P* ≤ 0.001, ***). n.t., not tested. (**C**) Pseudotyped particles bearing the indicated S proteins were preincubated (30 min, 37°C) with different dilutions of plasma derived from MVA-MERS-S vaccinated and infected (samples 1–3) or MERS-CoV seropositive (samples 4–9) camels before being inoculated onto Vero cells. At 16–18 h post-inoculation, pseudovirus entry was measured by quantifying luciferase activities in cell lysates. Cell entry in the absence of plasma was set as 0% inhibition. The serum dilution leading to a 50% reduction in S protein-driven cell entry (neutralizing titer 50, NT_50_) was calculated using a non-linear regression model. Samples that yielded NT_50_ values below 1 were considered negative and manually assigned an NT_50_ value of 1. Left panel: geometric mean NT_50_ values (geometric mean titers, GMT) from a single biological replicate (conducted with four technical replicates). The proportion of samples with detectable neutralizing activity is indicated. The dashed line indicates the lowest reciprocal dilution tested. Right panel: individual NT_50_ values per serum. Data points reflect neutralization by separate plasma samples but were connected to aid visual discrimination of NT50 values measured for individual S proteins. Statistical significance was assessed by Kruskal–Wallis analysis with Dunn’s multiple comparison test (*P* > 0.05, not significant [ns]; *P* ≤ 0.05, *; *P* ≤ 0.01, **; *P* ≤ 0.001, ***). n.t, not tested.

Having established that the S proteins studied mediated robust cell entry, we next investigated the temperature stability of S protein-bearing particles and their sensitivity to IFITM proteins and neutralizing antibodies. In order to address temperature stability, the particles harboring the S proteins were incubated for different time intervals at 33°C, 37°C, and 42°C and then added to target cells, followed by quantification of entry efficiency. No apparent differences in temperature sensitivity were noted (Fig. 3A). Similarly, entry of all S protein bearing particles was comparably inhibited by stable expression of IFITM1-3 proteins in target cells, with particles harboring the hemagglutinin and neuraminidase proteins of influenza A virus A/WSN/33 (known to be IFITM-sensitive) or the glycoprotein of Machupo virus (MACV-GPC, known to be IFITM-insensitive) serving as controls (Fig. 3B) (40). Finally, all S protein bearing particles were comparably neutralized by sera from MERS-CoV seropositive or vaccinated (with MVA encoding full-length MERS-CoV EMC S protein [41]) and infected camels (Fig. 3C; Table S1).

### Recent Arabian viruses are less sensitive to inhibition by soluble DPP4 due to mutation Q1020R

**Fig 4:**
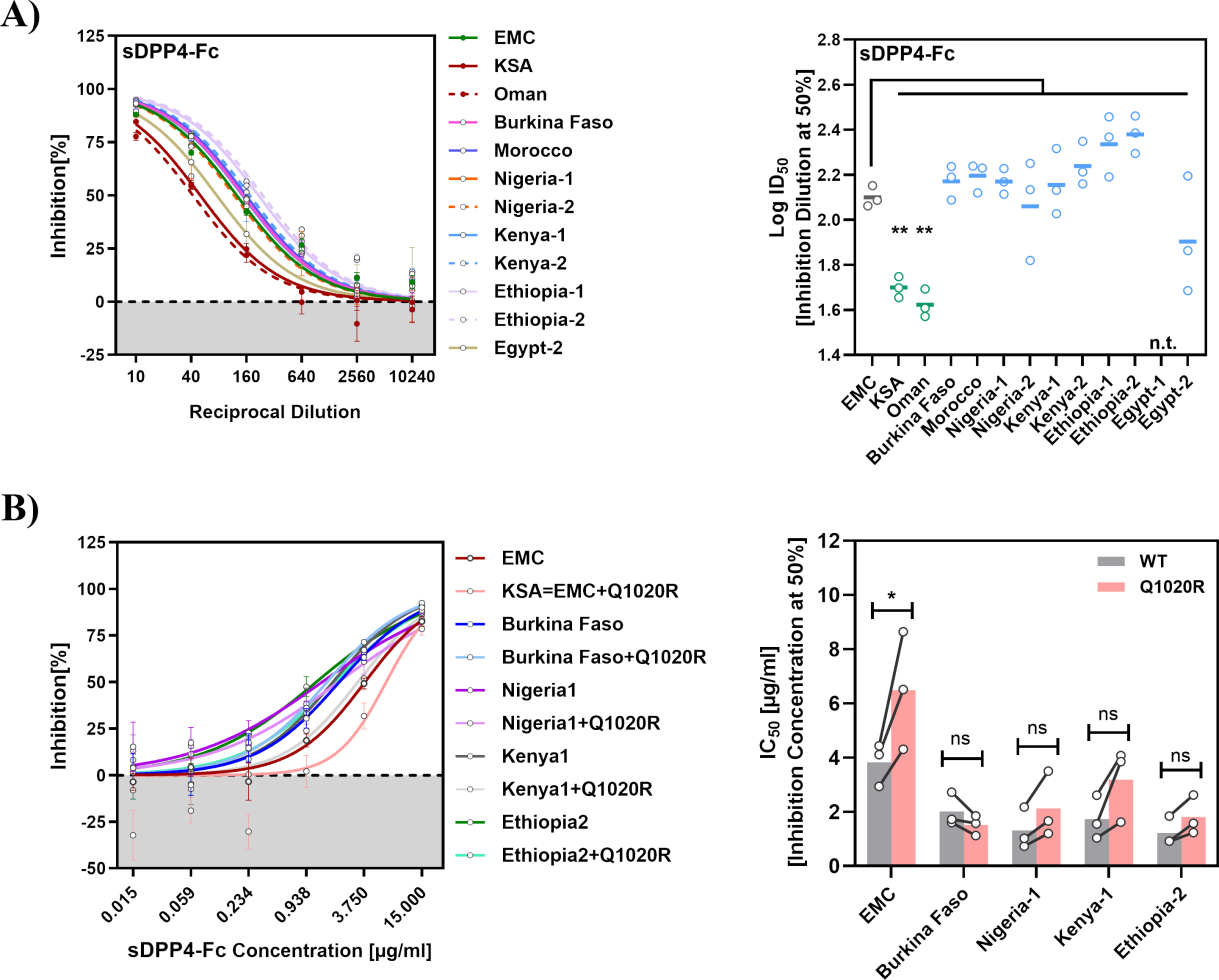
Particles pseudotyped with the S proteins of Arabian viruses are less sensitive to inhibition by sDPP4-Fc, and mutation Q1020R contributes to resistance. (**A**) Inhibition of entry by sDPP4-Fc. Particles harboring the indicated S proteins were preincubated at 37°C for 30 min with different dilutions of soluble human DPP4 harboring a C-terminal Fc-tag (sDPP4-Fc) before being inoculated onto Vero cells. At 16–18 h after inoculation, S-protein-driven cell entry was measured and normalized against particles incubated without sDPP4-Fc (= 0% inhibition). Presented are the average (mean) data ± SEM from three biological replicates (each performed with four technical replicates). The left graph shows dose-dependent inhibition of pseudovirus entry. The 50% inhibitory dilution (ID_50_) was calculated using a non-linear regression model and is presented on the right. Statistical significance of differences between particles bearing EMC S protein and the other S proteins tested was evaluated by one-way ANOVA with Dunnett’s posttest (*P* > 0.05, not significant [ns]; *P* ≤ 0.05, *; *P* ≤ 0.01, **; *P* ≤ 0.001, ***). n.t., not tested. (**B**) Impact of mutation Q1020R on sDPP4-Fc sensitivity. The experiment was conducted as in panel A, but commercial sDPP4-Fc was used, S proteins with and without mutation Q1020R were analyzed, and the 50% inhibitory concentration (IC_50_) is shown in the right panel. Statistical significance between wild type and Q1020R mutation was evaluated by two-way ANOVA followed by Šídák's posttest (*P* > 0.05, not significant [ns]; *P* ≤ 0.05, *; *P* ≤ 0.01, **; *P* ≤ 0.001, ***). n.t., not tested.

In humans, a soluble form of DPP4 (sDPP4) is naturally expressed in most bodily fluids (27–29), including lung fluid (42), and recombinant sDPP4 can block MERS-CoV cell entry (31, 32). Therefore, we also examined whether the S proteins studied exhibited different sensitivity to inhibition by human sDPP4. For this, we fused soluble DPP4 to the Fc portion of immunoglobulin G (sDPP4-Fc) and purified the fusion protein from 293T cells. Particles bearing the S proteins of viruses from Arabian camels were significantly less susceptible to inhibition by sDPP4-Fc as compared with particles bearing the S proteins from viruses found in African camels or EMC (Fig. 4A).

The S protein of the MERS-CoV isolate KSA-CAMEL-378, obtained from a camel from the Kingdom of Saudi Arabia (KSA), is identical at the amino acid level to S proteins of lineage five viruses responsible for recent human infections (8) but differs from EMC S protein only by mutation Q1020R (Fig. 1A). Furthermore, this mutation was present in the S proteins of the two Arabian but not the 10 African camel viruses studied here, and was also present in viruses responsible for the MERS outbreak in Korea in 2015. Therefore, we determined whether introducing Q1020R into the S proteins of EMC and selected viruses found in African camels reduced sensitivity to sDPP4-Fc. Introducing Q1020R into EMC S protein diminished inhibition by recombinant, commercial sDPP4-Fc (Fig. 4B), in agreement with the data obtained with self-made sDPP4-Fc (Fig. 4A). Furthermore, Q1020R reduced sDPP4-Fc sensitivity of particles bearing three out of the four S proteins from African camel viruses tested, although these effects were not statistically significant (Fig. 4B), suggesting that the magnitude of the effect of Q1020R on sDPP4 sensitivity can depend on the S protein context.

**Fig 5:**
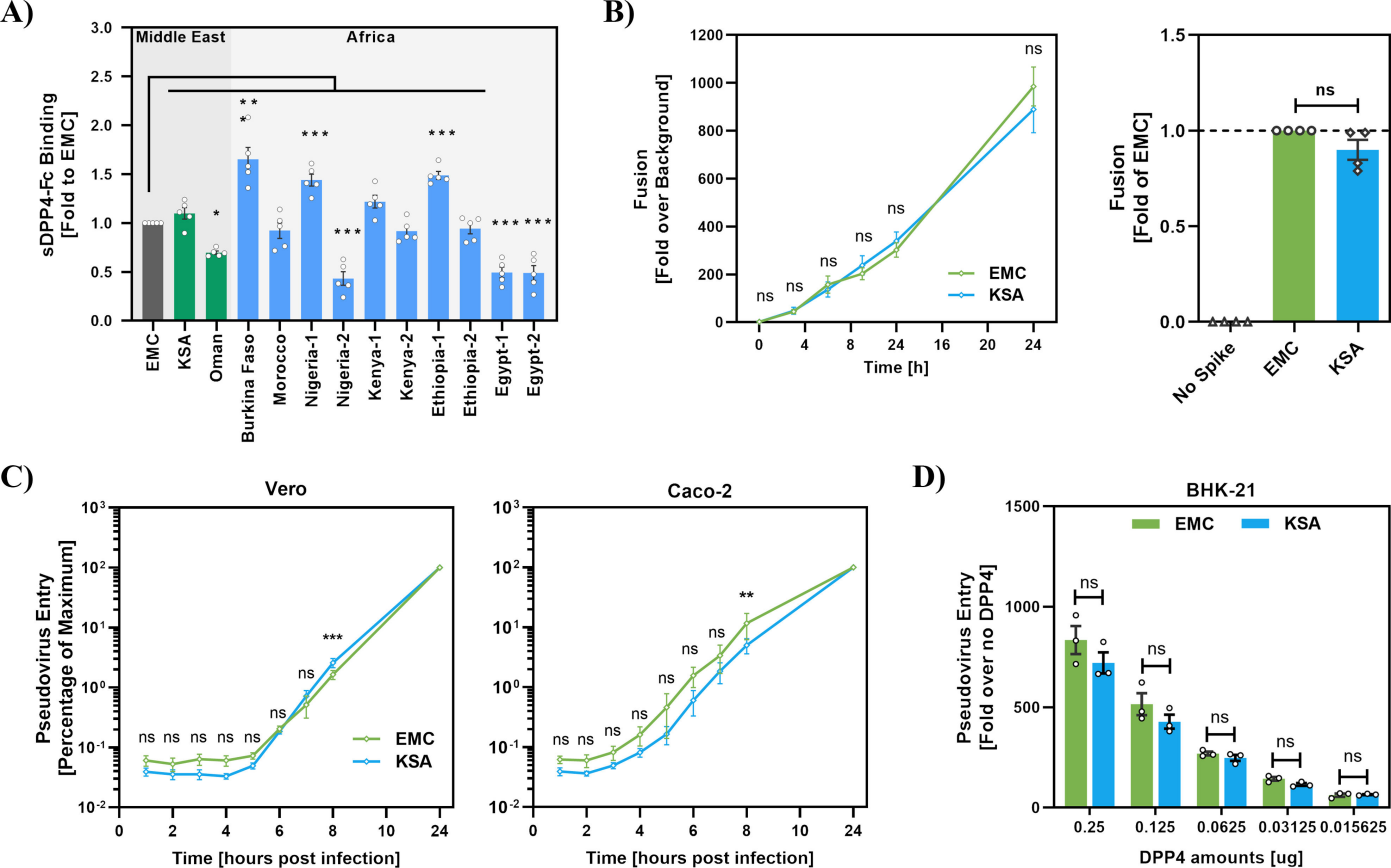
Mutation Q1020R does not reduce binding to sDPP4-Fc and does not alter the kinetics of S protein-driven cell–cell and virus-cell fusion. (**A**) For analysis of DPP4 binding, 293T cells transiently expressing the indicated S proteins (or no S protein as negative control) were incubated with sDPP4-Fc and binding analyzed by flow cytometry. Shown is the mean channel fluorescence from five biological replicates (each conducted with a single sample). For normalization, binding of sDPP4 to EMC S protein was set as 1, error bars represent the SEM. Statistical significance of differences between sDPP4-Fc binding to EMC S protein and the other S proteins studied was assessed by one-way analysis of variance (ANOVA) with Dunnett’s posttest (*P* > 0.05, not significant [ns]; *P* ≤ 0.05, *; *P* ≤ 0.01, **; *P* ≤ 0.001, ***). (**B**) To assess the kinetics of S protein-mediated cell-cell fusion, effector 293T cells were transfected with plasmids encoding the indicated S proteins together with the beta-galactosidase α-fragment and co-cultured with target 293T cells expressing human DPP4 and the complementary omega-fragment. Beta-galactosidase activity was quantified in cell lysates at the indicated time points. Data represent the means of four independent experiments performed in technical quadruplicates; error bars indicate SEM. Fusion dynamics (normalized to background from effector cells transfected without S protein) are shown in the left panel, while fusion efficiency at 24 h post co-culture (normalized to MERS-S EMC, set as 1) is shown in the right panel. Statistical significance was determined using two-way ANOVA with Dunnett’s posttest (left) and unpaired two-tailed Student’s *t*-test with Welch’s correction (right) (*P* > 0.05, ns; *P* ≤ 0.05, *; *P* ≤ 0.01, **; *P* ≤ 0.001, ***). (**C**) For analysis of the kinetics of virus-cell fusion, particles pseudotyped with the indicated S proteins were incubated with Vero and Caco-2 cells for the indicated time periods. After incubation, cells were lysed, and luciferase activity was measured. Transduction efficiency measured after 24 h (the maximum incubation time) was set to 100%. Data represent mean values from three biological replicates, each performed in technical quadruplicates; error bars indicate SEM. Statistical significance was determined by two-way ANOVA followed by Dunnett’s multiple comparison test (*P* > 0.05, ns; *P* ≤ 0.05, *; *P* ≤ 0.01, **; *P* ≤ 0.001, ***). (**D**) Ability to use low levels of DPP4 for cell entry. BHK-21 cells were transfected with serial dilutions of a plasmid encoding human DPP4 and then infected with pseudotyped particles bearing EMC or KSA S proteins. Luciferase activity was measured at 16–18 h post-infection and normalized to the signal in cells transfected with empty vector. Data represent mean values from three independent experiments performed in technical quadruplicates; error bars indicate SEM. Statistical significance was assessed by two-way ANOVA with Dunnett’s posttest (*P* > 0.05, ns; *P* ≤ 0.05, *; *P* ≤ 0.01, **; *P* ≤ 0.001, ***).

We next sought to obtain initial insights into why mutation Q1020R reduces sensitivity to sDPP4-Fc. We first investigated whether Q1020R alters the efficiency of sDPP4-Fc binding. However, when binding of sDPP4-Fc to S protein-expressing 293T cells was measured, no appreciable differences between EMC and KSA S proteins were observed (Fig. 5A). Similarly, no correlation between sDPP4-Fc binding efficiency (Fig. 5A) and susceptibility to sDPP4-Fc-mediated inhibition (Fig. 4A) was observed for the S proteins tested, suggesting that the relative sDPP4-Fc resistance associated with Q1020R might not be the result of reduced sDPP4-Fc binding. Second, we determined whether Q1020R increases the kinetics of S protein-driven cell-cell and virus-cell fusion, which, in turn, might account for increased resistance towards sDPP4-Fc. However, no differences in the kinetics of cell-cell and virus-cell fusion driven by EMC and KSA S protein were detected (Fig. 5B and C), and both S proteins were comparably able to use low levels of DPP4 for cell entry (Fig. 5D). The only difference between cell entry driven by EMC S protein (Q1020) and the KSA/Oman S proteins (both harbor R1020) was the reduced dependence of the latter S proteins on the activity of cathepsin B/L (Fig. 2B). However, it remains to be determined whether this phenotype is related to the reduced inhibition by sDPP4-Fc.

### Indirect evidence that plasma sDPP4 may inhibit MERS-CoV entry

**Fig 6:**
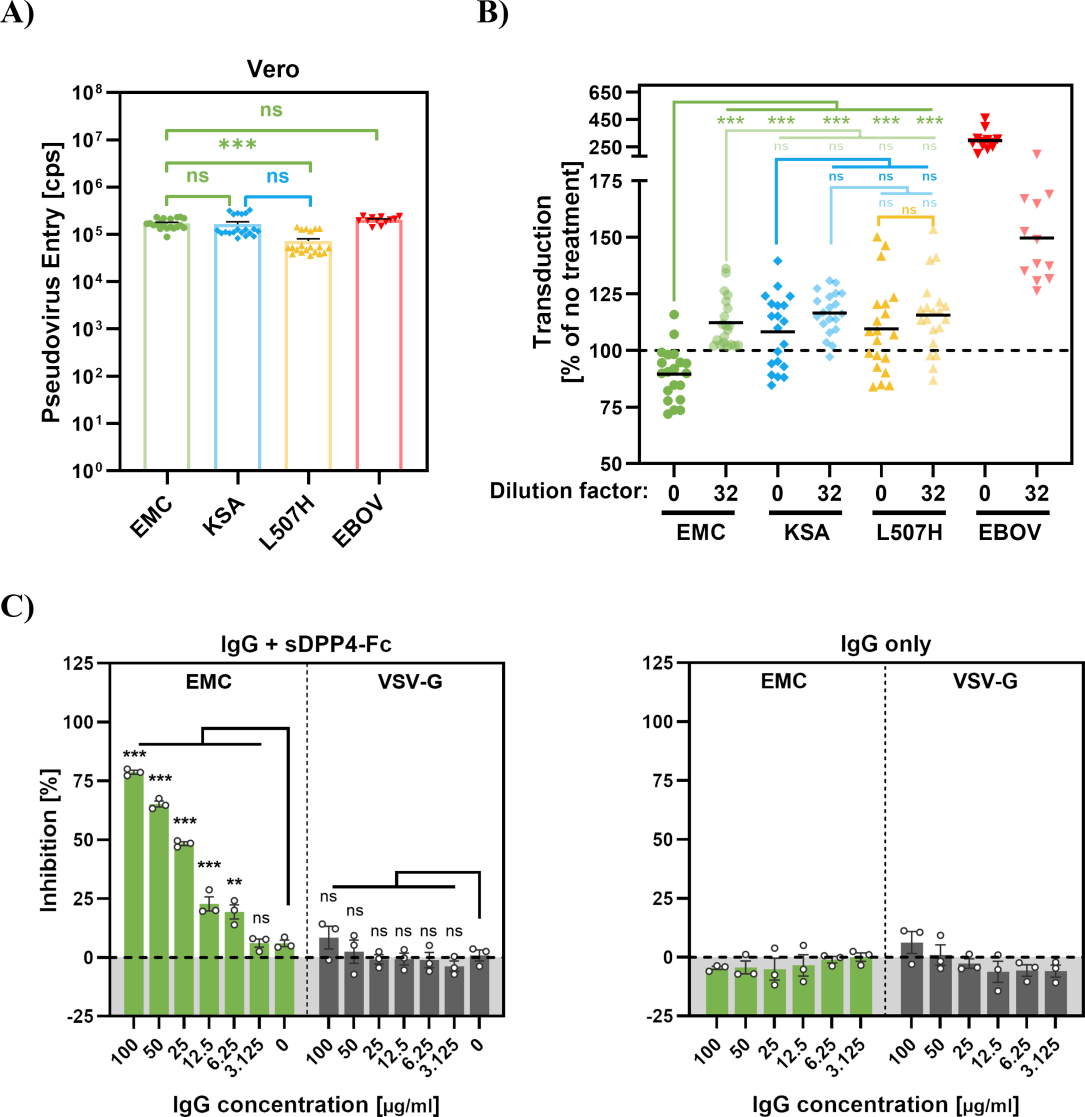
The Q1020R mutation confers resistance to entry inhibition by human plasma. (**A**) Pseudotype infectivity in the absence of plasma. Pseudotyped particles bearing the indicated glycoproteins were inoculated onto Vero cells that had not been treated with plasma. Cell entry was quantified at 16–18 h post-transduction by measuring pseudovirus-encoded luciferase activity in cell lysates. The results of 20 parallel infections carried out with technical quadruplicates are shown. Error bars represent SEM. Statistical significance was determined using a two-tailed Student’s *t*-test with Welch’s correction (*P* > 0.05, not significant [ns]; *P* ≤ 0.05, *; *P* ≤ 0.01, **; *P* ≤ 0.001, ***). (**B**) Pseudotyped particles expressing the indicated S proteins were preincubated (30 min, 37°C) with plasma (undiluted and 1:32 diluted) from healthy patients who had received mRNA vaccine against COVID-19 (*n* = 20). The mixtures were then inoculated onto Vero cells. At 16–18 h postinoculation, pseudovirus entry was quantified by measuring luciferase activity in cell lysates. As a negative control, pseudotyped particles bearing EBOV-GP were preincubated with a subset of plasma samples (*n* = 12) from the same cohort. The efficiency of entry in the absence of plasma treatment is shown in the left panel. Relative transduction (%) in the presence of plasma was calculated by normalizing the luciferase readings to those measured for particles in the absence of plasma (set as 100%) and is shown in the right panel. Results are based on a single experiment with four technical replicates. Statistical significance was determined using a two-tailed Student’s *t*-test with Welch’s correction (ns, *P* > 0.05; *, *P* ≤ 0.05; **, *P* ≤ 0.01; ***, *P* ≤ 0.001). Additional information is provided in Table S2. (**C**) To assess whether antibodies recognizing the Fc tag enhance the antiviral activity of sDPP4-Fc, pseudotyped particles bearing EMC-S or VSV-G were preincubated at 37°C for 30 min with sDPP4-Fc containing supernatants in the presence of increasing concentrations of human anti-Fc antibody, followed by addition to Vero target cells (left panel). At 16 h post-inoculation, pseudovirus entry was quantified by measuring luciferase activity in cell lysates and normalized to particles incubated without sDPP4-Fc (= 0% inhibition). To control for potential antibody-mediated effects, anti-Fc IgG was tested alone (without sDPP4-Fc) at the same concentrations. Luciferase activity in infected cells was quantified at 16–18 h post-infection and normalized to particles incubated without anti-Fc IgG (= 0% inhibition) (right panel). Two-way ANOVA with Dunnett’s posttest was used to determine statistical significance (*P* > 0.05, not significant [ns]; *P* ≤ 0.05, *; *P* ≤ 0.01, **; *P* ≤ 0.001, ***).

We next investigated whether sDPP4 in plasma inhibits MERS-CoV entry into cells. Previous studies reported sDPP4 concentrations in plasma that are close to IC_50_ values measured for recombinant protein (28, 32), but it has not been examined whether sDPP4 in plasma inhibits entry. For this, we employed human plasma samples and compared inhibition of entry driven by EMC and KSA S proteins. In addition, we analyzed EMC S protein harboring mutation L507H, which we found in a separate study to reduce DPP4 binding and susceptibility to inhibition by sDPP4-Fc (not shown). Further, we included the glycoprotein of Ebola virus (EBOV-GP) in our analyses since EBOV-GP-driven entry is independent of DPP4. Particles bearing the S proteins of EMC, KSA, EMC + L507H or EBOV-GP entered Vero cells with comparable efficiency in the absence of patient plasma (Fig. 6A). Notably, in the presence of plasma entry of particles bearing the EMC S protein but not KSA or EMC + L507H S proteins was significantly inhibited, and inhibition was not observed when plasma samples were diluted 1 to 32 (Fig. 6B). In contrast, plasma-augmented EBOV-GP-driven entry, in keeping with previous findings showing that lectins present in plasma like mannose-binding lectin (MBL) can augment filovirus infection (43–45). These results provide indirect evidence that sDPP4 in patient plasma can inhibit MERS-CoV entry.

We finally examined whether crosslinking of sDPP4-Fc dimers can increase antiviral activity. Indeed, preincubation of sDPP4-Fc with increasing amounts of Fc-specific antibody significantly augmented antiviral activity in a concentration-dependent manner (Fig. 6C). Although it remains to be determined whether sDPP4 multimerizes in patients, potentially due to certain autoantibodies (46, 47), our data suggest that multimerization likely increases anti-MERS-CoV activity.

## DISCUSSION

Our study demonstrates that the Q1020R mutation found in Arabian MERS-CoV confers resistance to soluble DPP4 (sDPP4). These findings support the concept that sDPP4 acts as a barrier to MERS-CoV infection in humans and that the Q1020R substitution enables viral variants to overcome this barrier. Specifically, sDPP4 levels in the Saudi Arabian population are reported to be lower compared with those in other ethnic groups (48), potentially providing less effective protection against infection, and the Q1020R mutation present in Arabian MERS-CoV variants may further weaken the sDPP4-based barrier to infection. Conversely, higher sDPP4 levels in individuals of African descent, compared with those of Arabian descent, together with the absence of the Q1020R mutation in African viruses, may help explain the absence of reported MERS cases in Africa.

The finding that mutation Q1020R reduces sensitivity to inhibition by sDPP4 is remarkable, considering that the S proteins analyzed here exhibited little differences in other aspects of the entry process. All S proteins were robustly incorporated into VSV particles, enabling meaningful functional comparisons, and exhibited comparable sensitivity to elevated temperatures, neutralizing antibodies, and expression of IFITM protein in target cells. Similarly, all S proteins allowed for comparable entry into multiple cell lines. A previous study reported moderately reduced Calu-3 lung cell entry for Burkina Faso and Nigeria-1 spikes as compared with EMC S protein (6). We did not observe this reduction in either the present study or our previous work (49), potentially due to differences in the pseudotyping systems employed (i.e., lentiviral [6] versus rhabdoviral [our studies] platforms). Evidence for reduced entry was also obtained for a chimeric MERS-CoV encoding the Burkina Faso spike (6), but viral protein expression was used as readout and confirmation in an entry-specific assay would be desirable. Finally, we noted that entry driven by the Kenya-1 and Kenya-2 S proteins showed increased sensitivity to a cathepsin L inhibitor, for at present unclear reasons, while the KSA and Oman S proteins exhibited increased resistance, as discussed below.

Recombinant sDPP4 inhibits MERS-CoV entry in cell culture and animal models (31, 32) and may provide an approach to antiviral therapy. In order for endogenous sDPP4 to impact MERS-CoV infection, it needs to be robustly expressed in relevant organs and bodily fluids. sDPP4 is present at high concentrations in plasma, where it may inhibit the hematogenous spread of MERS-CoV—a process likely to occur frequently in patients requiring hospitalization given the common detection of viremia in these patients (50–52)—and thereby contribute to protection against severe disease, as also discussed below. The IC_50_ values measured in the present study for sDPP4-Fc-mediated inhibition of viral entry (roughly 2–6 µg/mL) are higher than the sDPP4 concentrations found in the plasma of healthy donors (roughly 0.5–0.7 µg/mL) and MERS patients (28, 32), who show reduced sDPP4 levels as compared with healthy controls (48). However, sDPP4-Fc might exert less antiviral activity than endogenous sDPP4 since dimer formation of endogenous sDPP4 is driven by non-covalent interactions between DPP4 ectodomains (53), while dimerization of sDPP4-Fc is driven by the Fc portion—likely resulting in differences in the spatial orientation of the DPP4 ectodomain. Further, sDPP4 antiviral activity was more pronounced when sDPP4-Fc dimers were crosslinked, and it is conceivable that some DPP4 autoantibodies (46, 47) might drive multimerization and thereby augment antiviral activity. In sum, sDPP4 is present at high levels in plasma, and one can speculate that it may inhibit MERS-CoV infection, but this possibility has so far not been explored.

Our results suggest that sDPP4 in human plasma can indeed inhibit MERS-CoV infection—EMC S protein-driven entry was significantly inhibited by undiluted plasma, while this effect was not observed with S protein harboring Q1020R (KSA) or containing mutation L507H, which reduces DPP4 binding and susceptibility to inhibition by sDPP4 (not shown). Plasma samples were collected from COVID-19 mRNA vaccinated patients, but antibody responses induced by vaccination or SARS-CoV-2 infection do not or very rarely and inefficiently cross-neutralize MERS-CoV (54–56) and should not have impacted our results. Although analysis of sDPP4-depleted plasma is pending, our results provide, for the first time, indirect evidence that endogenous sDPP4 in plasma may inhibit MERS-CoV infection. This finding is noteworthy considering that a high percentage of MERS patients exhibit viremia, which likely promotes dissemination of the virus from the respiratory tract to other organs and has been associated with disease severity and mortality by several studies (1, 50, 52, 57).

It is at present unclear how mutation Q1020R bestows MERS-CoV with resistance against sDPP4. Q1020R has been detected in viruses from Arabian camels and MERS patients, and codon 1020 is under positive selection (58–61). The Q1020R mutation is located in the heptad repeat 1 (HR1) within the S2 subunit and decreases stability of the 6-helix bundle (58). Similarly, a nearby mutation, T1015N, alters S protein stability and increases entry efficiency (58). However, entry mediated by the KSA S protein (R1020) was only slightly more efficient than that facilitated by the EMC S protein (Q1020), and no differences in the kinetics of cell-cell and virus-cell fusion were observed. As a consequence, increased infectivity is unlikely to be responsible for resistance to sDPP4. Studies with murine coronaviruses have revealed that residues in HR1 or other functional elements in S2 can impact receptor engagement (62, 63). We found that Q1020R did not alter DPP4-binding efficiency or promote entry into cells expressing low levels of DPP4 at the plasma membrane, suggesting that Q1020R might not confer sDPP4 resistance by decreasing DPP4 binding. Finally, we note that KSA S protein-dependent entry was less susceptible to inhibition by MDL28170, which targets cathepsin B/L, than entry driven by EMC S protein, but it is currently unknown whether this phenotype is linked to reduced sensitivity to sDPP4.

In sum, our findings indicate that Arabian viruses harboring the Q1020R mutation are less susceptible to inhibition by human sDPP4 than viruses circulating in African camels. It will be interesting to determine in future studies whether sDPP4 is also generated in camels, exerts antiviral activity, and is differentially active against viruses bearing either Q or R at position 1020.

## MATERIALS AND METHODS

### Expression plasmids

Expression plasmids for DsRed (64), VSV-G (vesicular stomatitis virus glycoprotein) (65), MERS-S EMC (based on human betacoronavirus 2c EMC/2012 isolate) (37), Machupo virus glycoprotein (MACV-GPC, kindly provided by Michael Farzan) (66), influenza A virus strain A/WSN/33 (H1N1) hemagglutinin and neuraminidase (WSN-HA/NA) (67), human DPP4 (12), bat DPP4 (12), dromedary camel DPP4 (12) (kindly provided by Vincent Munster), and beta-galactosidase alpha and omega fragment (68) have been previously described. In addition, overlap extension PCR was utilized to generate expression vectors encoding S proteins from various regions: KSA (Kingdom of Saudi Arabia, KSA/Camel/378, GenBank: AHY22535.1), Oman (Oman, Camel/Oman_1_2015, GenBank: AQZ41296.1), Burkina Faso (Burkina Faso, camel/Burkina Faso/CIRAD-HKU785/2015, GenBank: MG923471.1), Morocco (Morocco, camel/Morocco/CIRAD-HKU213/2015, GenBank: MG923469.1), Nigeria-1 (Nigeria, camel/Nigeria/NV1657/2016, GenBank: MG923475.1), Nigeria-2 (Nigeria, camel/Nigeria/NV1405/2016, GenBank: AVN89376.1), Kenya-1 (Kenya, camel/Kenya/ 011/LOM/C20/F<1, GenBank: MK357909.1), Kenya-2 (Kenya, camel/Kenya/C1215/201, GenBank: AXP07345.1), Ethiopia-1 (Ethiopia, camel/Ethiopia/AAU-EPHI-HKU4412/2017, GenBank: MG923466.1), Ethiopia-2 (Ethiopia, camel/MERS/Amibara/118/2017, GenBank: MK564474), Egypt-1 (Egypt, camel/Egypt/NRCE-HKU205, GenBank: KJ477102.1), and Egypt-2 (Egypt, camel/Egypt/NRCE-HKU270, GenBank: KJ477103.2). For all S proteins, we also constructed versions with a C-terminal V5 tag for detection by immunoblotting. The mutation Q1020R was introduced into the S protein encoding plasmids by overlap extension PCR with overlapping primers containing the mutation. The expression vector for soluble human DPP4 was constructed as follows: First, the coding sequence for the signal peptide of human CD5 (amino acid [aa] sequence: MPMGSLQPLATLYLLGMLVASCLG) was fused to the coding sequence of the Fc portion of human immunoglobulin G (IgG, aa position: 99–330, taken from the pCG1-Fc plasmid (69) that was kindly provided by Georg Herrler. Of note, the IgG sequence harbors four variations [E99D T106P, P111L, A276T] compared to GenBank: AXN93647.1) followed by the extracellular region of human DPP4 (aa position: 29–765 of NCBI Reference Sequence: NP_001366533.1) by overlap extension PCR, including a linker sequence (aa sequence CLNLQACKLDIRGR) between IgG and DPP4. Finally, the resulting sequence was inserted into the pCG1 plasmid (kindly provided by Roberto Cattaneo) making use of BamHI and XbaI restriction sites. The integrity of all PCR-amplified sequences was verified by using a commercial sequencing service (Microsynth SeqLab).

### Cell culture

The following cell lines were used in the present study: Vero cells (African green monkey kidney, female; ATCC no. CRL-1586, RRID: CVCL_0603, kindly provided by Andrea Maisner), Huh-7 cells (human liver, male; JCRB no. JCRB0403; RRID: CVCL 0336, kindly provided by Thomas Pietschmann), BHK-21 (Syrian hamster, kidney cells; ATCC no. CCL-10; RRID: CVCL_10929, kindly provided by Georg Herrler), and 293T cells (human kidney, female; DSMZ no. ACC-635; RRID: CVCL_0063) were cultured in Dulbecco’s modified Eagle medium (DMEM, PAN-Biotech) supplemented with 10% fetal bovine serum (FBS, Biochrom), 1% 100 U/mL penicillin and 0.1 mg/ml streptomycin (pen/strep) (PAN-Biotech). 293T cells stably expressing human DPP4 N-terminally linked to DsRed (293T+DPP4) (37), IFITMs (293T-IFITM1, 293T-IFITM2, 293T-IFITM3) or chloramphenicol acetyltransferase (293T-CAT) (70) were cultured under the same conditions as the parental 293T cell line, with the addition of 0.5 µg/mL puromycin (Invivogen) for selection. Caco-2 cells (human colon, male; ATCC no. HTB-37; RRID: CVCL_0025) were cultured in minimum essential medium (MEM, Thermo Fisher Scientific) supplemented with 10% FBS, 1% pen/strep solution, 1% non-essential amino acid solution (PAA) and 1 mM sodium pyruvate (PAN-Biotech). Calu-3 cells (human lung, male; ATCC no. HTB-55; RRID: CVCL_0609, kindly provided by Stephan Ludwig) were cultured in DMEM/F-12 medium (Thermo Fisher Scientific) supplemented with 10% FBS, 1% pen/strep solution, 1% non-essential amino acid solution, and 1 mM sodium pyruvate. Calu-3 cells stably expressing the beta-galactosidase omega fragment (Calu3-Omega) were cultured in the same medium as the parent Calu3 cells, supplemented with puromycin (0.5 µg/mL). All cell lines were maintained in a humidified incubator at 37°C with 5% CO_2_. Cell lines were validated by short-tandem-repeat analysis, amplification and sequencing of a fragment of the cytochrome c oxidase gene, microscopic examination and/or assessment of growth characteristics. In addition, all cell lines were routinely tested for mycoplasma contamination. Transfection of 293T cells was performed by calcium-phosphate precipitation.

### Production of soluble human DPP4-Fc

An expression plasmid for human soluble DPP4 (sDPP4), linked to the Fc region of human immunoglobulin G, was transfected into 293T cells 24 h after seeding. The medium was changed after 16 h, and cells were incubated for an additional 32 h. The supernatant was collected on the fourth day after transfection, clarified from cells and debris by centrifugation (2,000 × *g*, 10 min, 4°C), and stored at 4°C. In addition, fresh medium was added to the cells, and the procedure was repeated on the fifth day after transfection, and the clarified supernatants were combined. Subsequently, sDPP4 from the remaining intact cells was harvested as follows. First, a small amount of fresh culture medium was added to the cells (2 mL/T75 flask), followed by two freeze-thaw cycles (2 h −80°C, 1 h 37°C) to break down the cellular membranes. Next, the cellular debris was pelleted by centrifugation, and the clarified supernatant was mixed with the combined supernatants of days 4 and 5. The material was subsequently loaded onto 50 kDa MWCO (molecular weight cut-off) Vivaspin Protein Concentrator columns (Sartorius) and centrifuged (4,000 × *g*, 4°C) until a 50-fold concentration factor was achieved. Next, sDPP4 was purified using NAb Protein A/G Spin Columns (Thermo Fisher Scientific, Cat: 89962) according to the manufacturer’s instructions. After purification, the sDPP4 was further concentrated 30-fold using Vivaspin Protein Concentrator columns (50 kDa MWCO), aliquoted, and stored at −80°C until use.

### Production of pseudotyped particles

Vesicular stomatitis virus pseudotype particles bearing the MERS-CoV S proteins under study, influenza A virus (WSN, subtype H1N1) hemagglutinin and neuraminidase (WSN-HA/NA), Machupo virus glycoprotein (MACV-GPC), VSV-G, or no viral glycoprotein (negative control, particles were produced in cells transfected with DsRed expression plasmid) were generated using a replication-deficient VSV vector that lacks the genetic information for VSV-G and instead expresses two reporter proteins, enhanced green fluorescent protein (eGFP) and firefly luciferase (FLuc) reporter genes, VSV*∆G-FLuc (kindly provided by Gert Zimmer) (71), using an established protocol (64). In brief, 24 h posttransfection with plasmid encoding the desired viral glycoprotein, 293T cells were infected with VSV*∆G-FLuc for 1 h at 37°C. Following this incubation, the inoculum was removed, and cells were washed in phosphate-buffered saline (PBS). To neutralize any residual input virus containing VSV-G, DMEM medium supplemented with anti-VSV-G antibody (culture supernatant from I1-hybridoma cells; ATCC no. CRL-2700) was then added (of note, the antibody was not added to the medium of cells transfected with VSV-G expression plasmid). The culture supernatant was harvested and centrifuged (4,000 × *g*, 10 min) to remove cellular debris after 16–18 h of incubation. The clarified supernatants were subsequently aliquoted and stored at −80°C.

### Immunoblot

In order to evaluate MERS-S protein incorporation into VSV pseudotype particles, pseudoviruses expressing wild-type (EMC) or mutant MERS-S proteins with a C-terminal V5 tag were concentrated by high-speed centrifugation (13,300 rpm, 90 min, 4°C) through a sucrose cushion (20% w/v sucrose in PBS). The concentrated particles were then lysed in an equal volume of 2× SDS-sample buffer (0.03 M Tris-HCl, 10% glycerol, 2% SDS, 5% β-mercaptoethanol, 0.2% bromophenol blue, 1 mM EDTA), heated at 96°C for 15 min, and subjected to SDS-PAGE. Proteins were transferred onto nitrocellulose membranes (Hartenstein), blocked by 5% skim milk in PBS-T (PBS containing 0.05% Tween-20) for 30 min, and incubated overnight at 4°C with primary antibodies targeting the V5 tag (1:500, Invitrogen, Cat: R960-25), or anti-VSV-M [23H12] antibody (1:1,000, Kerafast, Cat: EB0011). After incubation, the membranes were washed three times with PBS-T and subsequently incubated at room temperature for 1 h in 5% milk solution containing horseradish peroxidase-conjugated anti-mouse secondary antibodies (1:2,000, goat IgG anti-mouse IgG (H+L)-HRPO [Dianova, Cat: 115-035-045]). Following three additional washes with PBS-T, membranes were treated with a homemade chemiluminescent solution (0.1 M Tris-HCl [pH 8.6], 250 g/mL luminol, 0.1 mg/mL para-hydroxycoumaric acid, 0.3% hydrogen peroxide) and visualized using the ChemoCam imaging system with ChemoStar Professional software (Intas Science Imaging Instruments). To analyze S protein incorporation into VSV particles, protein bands were quantified using ImageJ software (version 1.53C, https://imagej.net/ij/ [72]). Total S protein signals (uncleaved, S0, and cleaved, S2) were normalized against their corresponding VSV-M signals and the resulting values were further normalized against the EMC S protein (set as 1).

### Cell-cell fusion assay

Effector 293T cells were transfected with expression plasmids for the respective S proteins (or an empty vector), along with a plasmid encoding the beta-galactosidase alpha fragment. At 24 h post-transfection, 293T cells were washed with PBS, resuspended in fresh medium, and then added on top of 80% confluent Calu3-Omega cells (target cells, which stably express the beta-galactosidase omega fragment). Following a co-cultivation period of 24 h, beta-galactosidase substrate (Gal-Screen, Thermo Fisher Scientific) was added, and the cells were incubated in the dark at room temperature for 90 min. Subsequently, luminescence was measured using a Hidex Sense Plate Luminometer (Hidex). Cell–cell fusion kinetics were analyzed using the same setup as described above, with the only modification that 293T cells transiently transfected with DPP4 and the beta-galactosidase omega fragment were used as target cells instead of Calu3-Omega cells. After 24 h of transfection, effector cells were washed, resuspended in medium, added to target cells, and fusion was assessed at the indicated time points.

### Analysis of spike protein-mediated cell entry

To evaluate cell tropism and host cell entry efficiency, target cells were seeded in 96-well plates and inoculated with equal volumes of pseudotype particles harboring different MERS-CoV S proteins, WSN-HA/NA, MACV-GPC, VSV-G or no viral glycoprotein (negative control). For experiments assessing receptor usage, BHK-21 cells were transfected with plasmids encoding the respective DPP4 orthologues prior to infection. After 16–18 h inoculation, transduction efficiency was analyzed by measuring the activity of virus-encoded luciferase in cell lysates. The culture medium was first removed, and cells were lysed in PBS supplemented with 0.5% Triton X-100 (Carl Roth) for 30 min at room temperature. Lysates were then transferred into white 96-well plates, mixed with a commercial luciferase substrate (Beetle-Juice, PJK), and luminescence was measured using a Hidex Sense Plate Luminometer (Hidex).

To assess inhibition of viral entry by protease inhibitors, target cells (Vero and Caco2) were pre-incubated for 1 h in a medium containing different concentrations of the corresponding inhibitor, either MDL28170 (cathepsin L inhibitor, Sigma-Aldrich) or camostat mesylate (TMPRSS2 inhibitor, Sigma-Aldrich), prior to inoculation with pseudovirus particles. Cells treated with medium containing the solvent (DMSO) instead of inhibitor served as control. To investigate the entry kinetics of S protein-bearing pseudotypes, the particles were added to Vero or Caco-2 cells and incubated for different time intervals. After incubation, the cells were lysed, and luciferase activity was measured.

### Evaluation of sDPP4-mediated inhibition of S protein-driven cell entry

To analyze the inhibitory effect of sDPP4 on S protein-driven cell entry, S protein bearing particles were pre-incubated at 37°C for 30 min with different dilutions of self-made sDPP4-Fc or different concentrations of commercial sDPP4-Fc (Biozol, Cat: BSS-BS-47084P) prior to being added to Vero cells. Particles incubated in medium without sDPP4-Fc served as control. Transduction efficiency was assessed at 16-18 h postinoculation by measuring luciferase activity in cell lysates, as described above.

### Neutralization assay

For neutralization assays, pseudovirus particles were pre-incubated (30 min, 37°C) with different dilutions of camel plasma (1:400, 1:1,600, 1:6,400, 1:25,600, 1:102,400) or human plasma (undiluted, 1:32) before being inoculated onto Vero cells. Of note, pseudovirus particles exposed to medium without plasma served as controls. Transduction efficiency was assessed 16–18 h postinoculation by measuring luciferase activity in cell lysates, as described above.

### Temperature stability

To evaluate the thermostability of the indicated spike proteins, pseudotyped particles were pre-incubated at 33°C, 37°C, or 42°C for various durations (0, 3, 6, 12, 24, and 48 h) before being added to Vero cells. The luciferase activity in cell lysates was measured as described above and normalized to initial entry efficiency (0 h pre-incubation).

### DPP4 binding efficiency

To assess the binding of sDPP4-Fc to the S protein, 293T cells were seeded in 6-well plates and transfected with plasmids expressing the respective S protein or empty vector (serving as a negative control). The culture medium was replaced at 24 h post-transfection. At 48 h post-transfection, the culture medium was removed, and the cells were washed, resuspended in PBS, and pelleted by centrifugation (600 × *g*, 5 min, 4°C). After aspirating the supernatant, the cells were rinsed in PBS-B (PBS containing 1% bovine serum albumin [BSA; Carl Roth]) and pelleted again. Subsequently, cell pellets were resuspended in PBS-B containing sDPP4-Fc (1:200; ACROBiosystems, Cat: DP4-H5266) and incubated at 4°C for 1 h using a Rotospin rotator disk (IKA). Next, cells were pelleted, resuspended in PBS-B containing anti-human AlexaFluor-488-conjugated antibody (1:200; Thermo Fisher Scientific), and rotated again for 1 h at 4°C. Finally, the cells were washed with PBS-B, resuspended in PBS-B, and then analyzed via flow cytometry using an ID7000 Spectral Cell Analyzer (Sony Biotechnology, San Jose, CA, USA).

### Camel and human plasma samples

No sampling of camels was conducted for the present study, previously published plasma samples were analyzed (73, 74). Information on the camel plasma samples is provided in Table S1. Human plasma samples were obtained from COVID-19-vaccinated donors (*n* = 20; median age = 53 years; male to female ratio 9:11). Detailed characteristics of the donors are listed in Table S2. All plasma samples were heat-inactivated (56°C, 30 min) before use in neutralization experiments.

### Data analysis

Data analysis was conducted using Microsoft Excel (as part of the Microsoft Office software package, version 2016, Microsoft Corporation, Redmond, WA, USA) and GraphPad Prism version 8.3.0 (GraphPad Software, San Diego, CA, USA). Statistical analysis included one-way analysis of variance (ANOVA) with Dunnett’s posttest (incorporation efficiency, fusion assay, cell entry mediated by S protein, inhibition of entry by inhibitors or sDPP4, DPP4 binding by FACS), two-way ANOVA with Dunnett’s posttest (receptor usage, IFITM susceptibility, impact of mutation Q1020R on DPP4 sensitivity, the kinetics of cell-cell and virus-cell fusion driven by mutation Q1020R, Q1020R-mediated entry using low DPP4 levels), Kruskal–Wallis analysis with Dunn’ multiple comparison test (camel plasma neutralization assay), or two-tailed Student’s t test with Welch correction (S-mediated entry inhibited by human plasma). The plasma dilutions that result in half-maximal inhibition (neutralizing titer 50, NT_50_), sDPP4-Fc dilution factor that results in 50% inhibition (inhibitory dilution 50%, ID_50_), and the concentration of sDPP4-Fc, camostat, or MDL28170 that achieves 50% inhibition (inhibitory concentration 50%, IC_50_) were calculated using a non-linear regression model. Only *P*-values of 0.05 or lower were considered statistically significant (*P* > 0.05, not significant [ns]; *P* ≤ 0.05, *; *P* ≤ 0.01, **; *P* ≤ 0.001, ***).

## Data Availability

All data generated during this study are included in this article. GenBank accession numbers cited refer to sequences previously deposited by others and are publicly available through the National Center for Biotechnology Information (NCBI) database.

## SUPPLEMENTAL MATERIAL

Figure S1 jvi.00282-26-s0001.tif Location of *S protein* mutations for the MERS-CoV isolates under study.

Supplemental legend jvi.00282-26-s0002.docx Legend for Figure S1.

Table S1 jvi.00282-26-s0003.pdf Information on camel plasma samples.

Table S2 jvi.00282-26-s0004.pdf Information on human plasma samples.

ASM does not own the copyrights to Supplemental Material that may be linked to, or accessed through, an article. The authors have granted ASM a non-exclusive, world-wide license to publish the Supplemental Material files. Please contact the corresponding author directly for reuse.
