# Supplementary figures and images for: Q1020R in the spike proteins of MERS-CoV from Arabian camels confers resistance against soluble human DPP4
Source: J Virol. 2026 Apr 6;100(5):e00282-26. doi: 10.1128/jvi.00282-26 (PMC13185592; doi:10.1128/jvi.00282-26)

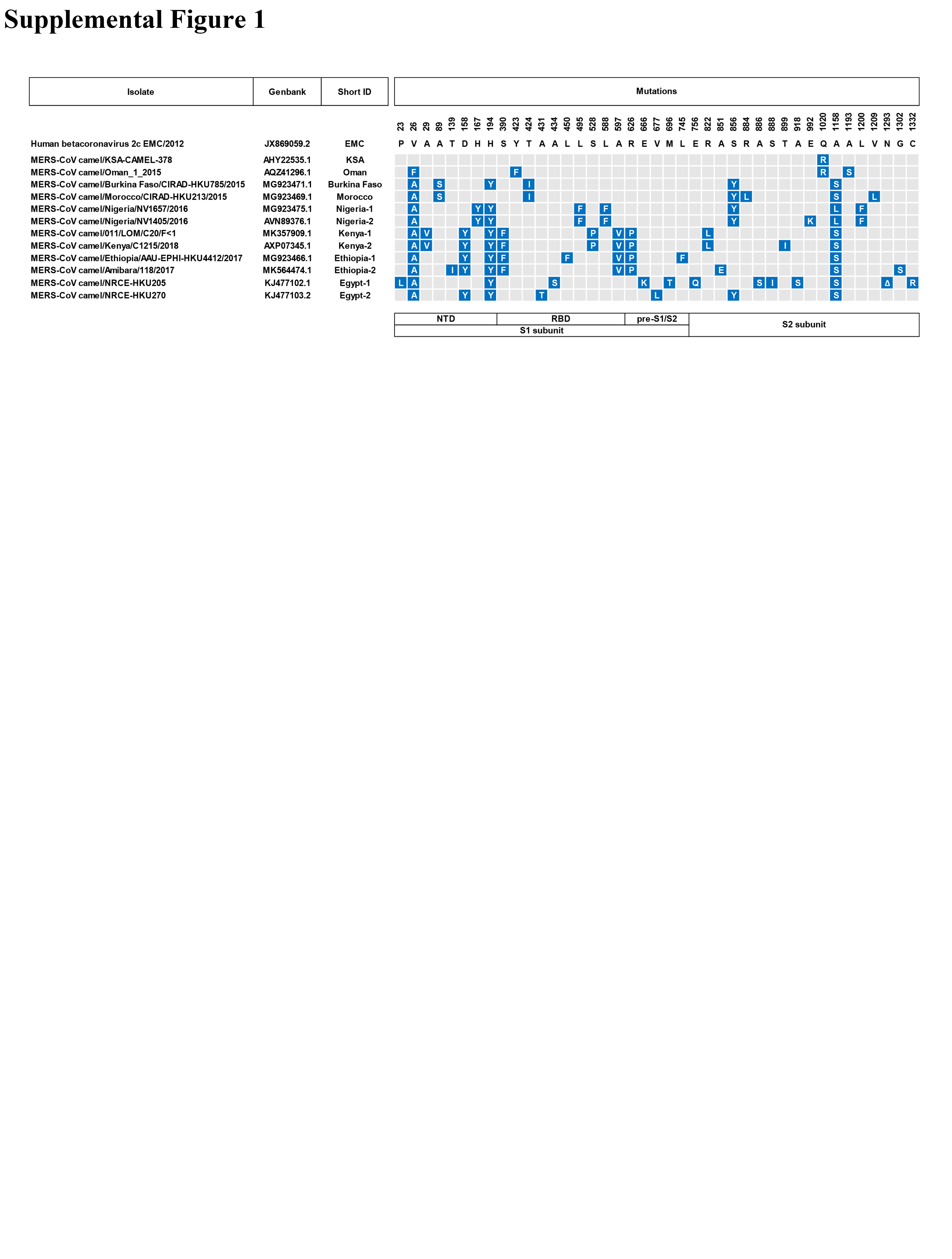

Supplement: Figure S1 — Location of S protein mutations for the MERS-CoV isolates under study. [file jvi.00282-26-s0001.tif]
