# Supplementary material for: Q1020R in the spike proteins of MERS-CoV from Arabian camels confers resistance against soluble human DPP4
Source: J Virol. 2026 Apr 6;100(5):e00282-26. doi: 10.1128/jvi.00282-26 (PMC13185592; doi:10.1128/jvi.00282-26)
Supplement: Supplemental legend — Legend for Figure S1. [file jvi.00282-26-s0002.docx]

**Legend to Supplemental Figure 1.**

**Supplemental Figure 1.** Location of S protein mutations for the MERS-CoV isolates under study. Indicated are the isolate names with GenBank identifiers and short names used in this study. Numbers above the mutations chart indicate amino acid positions (numbering according to EMC-S) with the respective residue in EMC-S stated. Grey boxes indicate no mutation, while blue boxes with letters indicate isolate-specific mutations (the delta sign indicates a deletion in the Egypt-1 S protein). Abbreviations: NTD, N-terminal domain; RBD, receptor-binding domain; pre-S1/S2, region between the RBD and the S2’ cleavage site.
