# Supplementary material for: Q1020R in the spike proteins of MERS-CoV from Arabian camels confers resistance against soluble human DPP4
Source: J Virol. 2026 Apr 6;100(5):e00282-26. doi: 10.1128/jvi.00282-26 (PMC13185592; doi:10.1128/jvi.00282-26)
Supplement: Table S1 — Information on camel plasma samples. [file jvi.00282-26-s0003.pdf]

**Supplemental Table 1, related to Figure 3C: Information on camel plasma samples**

| Plasma No. | Species | Location | Vaccinated or infected | Reference |
|---|---|---|---|---|
| 1 | Camel | Arabian peninsula | Infected, MVA-MERS-S vaccinated | [1,2] |
| 2 | Camel | Arabian peninsula | Infected, MVA-MERS-S vaccinated | [1,2] |
| 3 | Camel | Arabian peninsula | Infected, MVA-MERS-S vaccinated | [1,2] |
| 4 | Camel | Arabian peninsula (UAE) | Seropositive for MERS-CoV | [3] |
| 5 | Camel | Arabian peninsula (UAE) | Seropositive for MERS-CoV | [3] |
| 6 | Camel | Arabian peninsula (UAE) | Seropositive for MERS-CoV | [3] |
| 7 | Camel | East Africa (Kenya) | Seropositive for MERS-CoV | [4] |
| 8 | Camel | East Africa (Kenya) | Seropositive for MERS-CoV | [4] |
| 9 | Camel | East Africa (Kenya) | Seropositive for MERS-CoV | [4] |

**Supplemental References**


[1] Meyer Zu Natrup C, Schunemann LM, Saletti G, et al. MERS-CoV–Specific T-Cell Responses in Camels after Single MVA-MERS-S Vaccination. Emerg Infect Dis. 2023;29(6):1236-1239.

[2] Volz A, Kupke A, Song F, et al. Protective Efficacy of Recombinant Modified Vaccinia Virus Ankara Delivering Middle East Respiratory Syndrome Coronavirus Spike Glycoprotein. J Virol. 2015;89(16):8651-6.

[3] Meyer B, Muller MA, Corman VM, et al. Antibodies against MERS coronavirus in dromedary camels, United Arab Emirates, 2003 and 2013. Emerg Infect Dis. 2014;20(4):552-9.

[4] Corman VM, Jores J, Meyer B, et al. Antibodies against MERS coronavirus in dromedary camels, Kenya, 1992-2013. Emerg Infect Dis. 2014;20(8):1319-22.
