# Supplementary material for: Q1020R in the spike proteins of MERS-CoV from Arabian camels confers resistance against soluble human DPP4
Source: J Virol. 2026 Apr 6;100(5):e00282-26. doi: 10.1128/jvi.00282-26 (PMC13185592; doi:10.1128/jvi.00282-26)
Supplement: Table S2 — Information on human plasma samples. [file jvi.00282-26-s0004.pdf]

Supplemental Table 2, related to Figure 6A: Information on human plasma samples

#	Sex	Age	Sampling	Infection status (SARS-CoV-2)	Number of COVID-19 vaccinations	Last vaccine	Last vaccination	Time between last vaccination and sampling [d]	Time between last vaccination and sampling [weeks]	SARS-CoV-2 S1 IgG (BAU/mL)	NT50 (B.1)
9469	F	44	11/2/2022	Positive	4	BA.4/BA.5-adapted mRNA vaccine (BioNTech/Pfizer)	9/30/2022	33	4.7	11589.0	8813
9471	M	40	11/2/2022	Positive	4	BA.4/BA.5-adapted mRNA vaccine (BioNTech/Pfizer)	9/30/2022	33	4.7	7462.0	4651
9472	F	54	11/2/2022	Negative	4	BA.4/BA.5-adapted mRNA vaccine (BioNTech/Pfizer)	9/30/2022	33	4.7	2720.1	694
9473	F	58	11/2/2022	Positive	4	BA.4/BA.5-adapted mRNA vaccine (BioNTech/Pfizer)	9/30/2022	33	4.7	10399.7	3810
9474	F	54	11/2/2022	Negative	4	BA.4/BA.5-adapted mRNA vaccine (BioNTech/Pfizer)	9/30/2022	33	4.7	2628.6	1007

9477	M	52	11/2/2022	Negative	4	BA.4/BA.5-adapted mRNA vaccine (BioNTech/Pfizer)	9/30/2022	33	4.7	2374.5	1171
9478	F	42	11/2/2022	Positive	4	BA.4/BA.5-adapted mRNA vaccine (BioNTech/Pfizer)	9/30/2022	33	4.7	6435.4	3604
9479	M	62	11/2/2022	Negative	4	BA.4/BA.5-adapted mRNA vaccine (BioNTech/Pfizer)	9/30/2022	33	4.7	5576.4	973
9480	M	54	11/2/2022	Positive	4	BA.4/BA.5-adapted mRNA vaccine (BioNTech/Pfizer)	10/19/2022	14	2.0	6435.4	3392
9482	M	39	11/2/2022	Positive	4	BA.4/BA.5-adapted mRNA vaccine (BioNTech/Pfizer)	9/30/2022	33	4.7	8427.7	2314
9485	M	68	11/2/2022	Negative	4	BA.4/BA.5-adapted mRNA vaccine (BioNTech/Pfizer)	10/7/2022	26	3.7	2638.8	448
9486	F	52	11/2/2022	Negative	4	BA.4/BA.5-adapted mRNA vaccine (BioNTech/Pfizer)	10/10/2022	23	3.3	8519.2	4148

9488	M	54	11/2/2022	Negative	4	BA.4/BA.5-adapted mRNA vaccine (BioNTech/Pfizer)	10/7/2022	26	3.7	2643.8	1586
9489	F	58	11/2/2022	Negative	5	BA.1-adapted mRNA vaccine (BioNTech/Pfizer)	9/22/2022	41	5.9	3370.6	1818
9490	M	49	11/2/2022	Positive	4	BA.4/BA.5-adapted mRNA vaccine (BioNTech/Pfizer)	10/7/2022	26	3.7	13794.8	11839
9491	F	46	11/2/2022	Negative	4	BA.4/BA.5-adapted mRNA vaccine (BioNTech/Pfizer)	9/30/2022	33	4.7	4773.4	1294
9492	M	50	11/2/2022	Positive	4	BA.4/BA.5-adapted mRNA vaccine (BioNTech/Pfizer)	9/30/2022	33	4.7	8371.8	5320
9493	F	61	11/2/2022	Negative	4	BA.4/BA.5-adapted mRNA vaccine (BioNTech/Pfizer)	10/5/2022	28	4.0	4819.1	2528
9495	M	54	11/2/2022	Negative	4	BA.4/BA.5-adapted mRNA vaccine (BioNTech/Pfizer)	9/30/2022	33	4.7	2862.4	702
9496	M	40	11/2/2022	Negative	4	BA.4/BA.5-adapted mRNA	9/30/2022	33	4.7	8193.9	5787

						vaccine (BioNTech/Pfizer)					
